# Review of applications of deep learning in veterinary diagnostics and animal health

**DOI:** 10.3389/fvets.2025.1511522

**Published:** 2025-03-12

**Authors:** Sam Xiao, Navneet K. Dhand, Zhiyong Wang, Kun Hu, Peter C. Thomson, John K. House, Mehar S. Khatkar

**Affiliations:** ^1^Faculty of Science, Sydney School of Veterinary Science, The University of Sydney, Camden, NSW, Australia; ^2^School of Computer Science, The University of Sydney, Darlington, NSW, Australia; ^3^School of Science, Edith Cowan University, Joondalup, WA, Australia; ^4^School of Animal and Veterinary Sciences, University of Adelaide, Roseworthy Campus, Roseworthy, SA, Australia

**Keywords:** machine learning, deep learning, neural networks, computer vision, automated disease detection, image analysis, digital pathology

## Abstract

Deep learning (DL), a subfield of artificial intelligence (AI), involves the development of algorithms and models that simulate the problem-solving capabilities of the human mind. Sophisticated AI technology has garnered significant attention in recent years in the domain of veterinary medicine. This review provides a comprehensive overview of the research dedicated to leveraging DL for diagnostic purposes within veterinary medicine. Our systematic review approach followed PRISMA guidelines, focusing on the intersection of DL and veterinary medicine, and identified 422 relevant research articles. After exporting titles and abstracts for screening, we narrowed our selection to 39 primary research articles directly applying DL to animal disease detection or management, excluding non-primary research, reviews, and unrelated AI studies. Key findings from the current body of research highlight an increase in the utilisation of DL models across various diagnostic areas from 2013 to 2024, including radiography (33% of the studies), cytology (33%), health record analysis (8%), MRI (8%), environmental data analysis (5%), photo/video imaging (5%), and ultrasound (5%). Over the past decade, radiographic imaging has emerged as most impactful. Various studies have demonstrated notable success in the classification of primary thoracic lesions and cardiac disease from radiographs using DL models compared to specialist veterinarian benchmarks. Moreover, the technology has proven adept at recognising, counting, and classifying cell types in microscope slide images, demonstrating its versatility across different veterinary diagnostic modality. While deep learning shows promise in veterinary diagnostics, several challenges remain. These challenges range from the need for large and diverse datasets, the potential for interpretability issues and the importance of consulting with experts throughout model development to ensure validity. A thorough understanding of these considerations for the design and implementation of DL in veterinary medicine is imperative for driving future research and development efforts in the field. In addition, the potential future impacts of DL on veterinary diagnostics are discussed to explore avenues for further refinement and expansion of DL applications in veterinary medicine, ultimately contributing to increased standards of care and improved health outcomes for animals as this technology continues to evolve.

## Introduction

The field of artificial intelligence (AI) involves the development of computer systems that can emulate human like problem-solving abilities. AI systems are increasingly demonstrating proficiency across a wide range of sectors. These AI methods have been widely studied and applied to improve many aspects of a diverse range of disciplines in human medicine, such as drug development and delivery, patient monitoring, surgery, diagnostic imaging, screening, etc. (1). Numerous studies have consistently demonstrated that many AI models are at least as good as healthcare experts and specialists in performing some of the tasks, which they are designed to do, and even surpass the performance of the experts in some cases (2). This highlights the transformative capabilities of AI in addressing complex healthcare challenges.

### Machine learning and deep learning in veterinary medicine

Machine learning (ML) is a core approach in artificial intelligence (AI) that enables computers to learn from data and make predictions without explicit programming. ML encompasses two broad categories: traditional ML and deep learning (DL). Traditional ML methods, such as support vector machines (SVM), k-nearest neighbours (k-NN), and random forests, have been widely applied in livestock health, including oestrus and calving prediction, lameness detection, and disease monitoring (3–5). While these methods have demonstrated success in precision livestock farming and veterinary diagnostics (6), they often require manual feature engineering, which limits their scalability for complex data.

**Figure 1:**
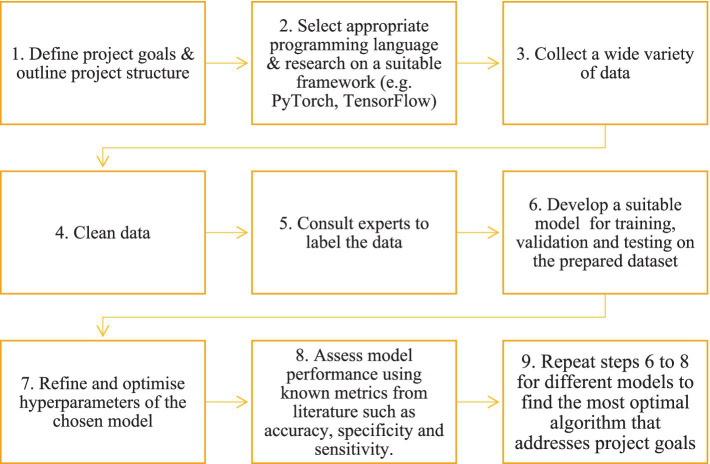
Example workflow of the development of a deep learning system.

DL, on the other hand, is a subset of AI that is better suited for processing large amounts of complex data compared to ML with the additional costs of requiring higher computation power. The key difference between traditional ML and DL lies in feature engineering and model complexity. Traditional ML models often require manual feature engineering to extract relevant information from the data. In contrast, DL models, based on neural networks, automatically learn relevant features from the data during the training process, reducing the need for extensive manual intervention (7). DL involves the use of various types of neural networks with many layers, hence the term “deep.” These models are inspired by the structure and function of the neuron connections in the brain and are capable of learning from complex, high-dimensional data by breaking it down into different layers or representations for analysis. Through DL, machines can now achieve a more nuanced approach to detecting trends in data, leading to accurate predictions and insights across numerous applications. DL technology has been a driving force behind many recent advancements in AI, including speech recognition, image recognition, and natural language processing (8). Figure 1 provides a brief general overview of the key stages involved in the workflow process of the development of a DL model.

Among DL architectures, convolutional neural networks (CNNs) are the frequently used for image analysis in veterinary medicine. CNNs excel at processing visual data by learning spatial hierarchies of features, making them highly effective for disease detection, classification, and segmentation in medical imaging. The general CNN architecture consists of:

- Convolutional layers, which apply filters to extract essential patterns such as edges, textures, and structures from input images.
- Pooling layers, which reduce the spatial dimensions of feature maps, enhancing computational efficiency while preserving critical features.
- Fully connected layers, which integrate extracted features for classification or regression tasks (9, 10).

CNNs form the backbone of many advanced DL architectures, such as ResNet, EfficientNet, and Inception, which have been successfully applied in veterinary diagnostics for disease classification and prognosis prediction. These architectures continue to drive progress in AI-assisted veterinary medicine (11).

Deep learning has been extensively applied in human medicine for diagnosis, treatment planning, and disease monitoring, leading to improved patient outcomes and cost reductions (12, 13). However, its application in veterinary medicine remains in its early stages (6). This review examines the current state of DL applications in veterinary diagnostics and animal health, highlighting key advancements, challenges, and potential future directions in the field.

## Methods

**Figure 2:**
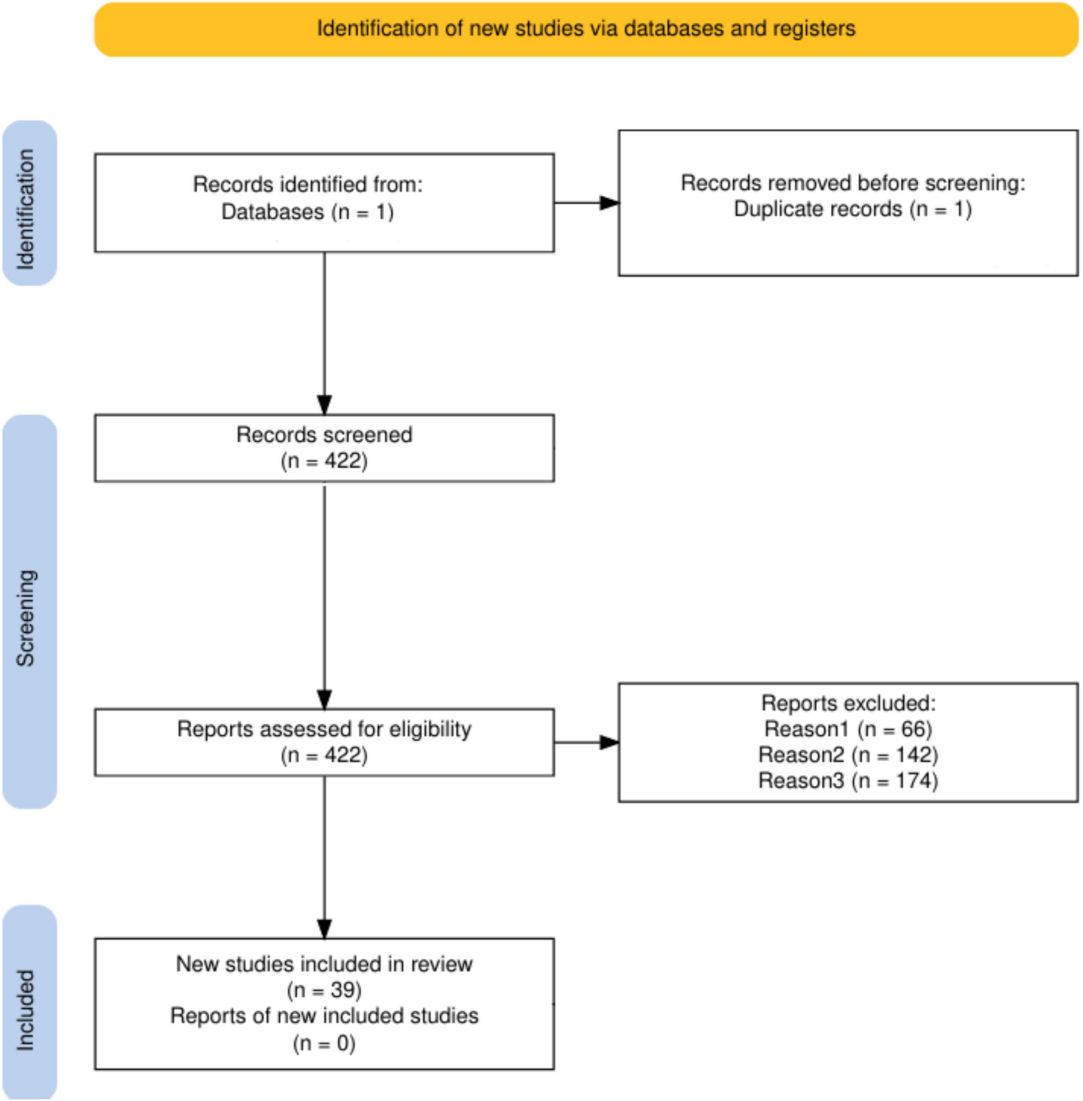
PRISMA flow diagram.

We conducted this systematic review following the Preferred Reporting Items for Systematic Reviews and Meta-Analyses (PRISMA) guidelines (Figure 2). The PRISMA 2020 guidelines provide a universal framework that contains a 27-element checklist and a flow diagram to ensure the comprehensive documentation of the review process, from literature search and study selection to data extraction and synthesis (14). The search query included the terms ‘Deep learning’ and ‘Veterinary’ within the PubMed database, aiming to identify literature focused on the application of deep learning (DL) techniques in veterinary medicine. This search yielded 422 relevant articles. The titles and abstracts of these articles were exported as a CSV file for further examination, and only primary research articles were retained. A total of 66 non-primary articles, including those related to veterinary curriculum, review articles, and books or book chapters mentioning AI, were excluded. Abstracts of the remaining articles were carefully reviewed to confirm that they involved the use of deep neural networks for animal disease diagnostics. Articles that mislabelled simple neural networks or ML techniques as DL, used DL only for preprocessing, object detection, or segmentation, or focused on animal models for human medicine were excluded, totalling 142 articles. Additionally, 174 articles related to human medicine and one duplicate were removed. Finally, 39 articles met the inclusion criteria (Table 1). Only full-text articles available via open access or the University of Sydney’s institutional access were included in this review.

**Table 1:** Applications of deep learning in veterinary medicine.

Title	Year	DL Technique	Sample size	Type of animal	Type of data/Modality	Evaluation metric	Reference
Using machine learning to classify image features from canine pelvic radiographs: evaluation of partial least squares discriminant analysis and artificial neural network models	2013	ANN	256	Canine	Radiograph	Classification error: 0.089 Sensitivity: 0.86 Specificity: 1	(36)
A methodological approach for deep learning to distinguish between meningiomas and gliomas on canine MR-images	2018	CNN (GoogLeNet)	80	Canine	MRI	Accuracy Post-contrast T1: 0.94 Pre-contrast T1: 0.91 T2 images: 0.90	(24)
Use of transfer learning to detect diffuse degenerative hepatic diseases from ultrasound images in dogs: A methodological study	2018	CNN (AlexNet)	52	Canine	Ultrasound	AUROC: 0.91 Sensitivity: 1 Specificity: 0.83	(24)
Using Convolutional Neural Networks for Determining Reticulocyte Percentage in Cats	2018	CNN	1,046	Feline	Microscope slide image	Accuracy: 0.987	(40)
Prediction of radiographic abnormalities by the use of bag-of-features and convolutional neural networks	2018	CNN	7,138	Canine	Radiograph	Accuracy: 0.929–0.969 Sensitivity: 0.921–1 Specificity: 0.938–0.96	(16)
A combined deep learning gru-autoencoder for the early detection of respiratory disease in pigs using multiple environmental sensors	2018	RNN (Gru)	Pig farms across Europe (unspecified)	Swine	Sensor data	Precision: 0.909 Recall: 0.909	(62)
Predicting early risk of chronic kidney disease in cats using routine clinical laboratory tests and machine learning	2019	RNN	106,251	Feline	Electric health record	Sensitivity: 0.907 Specificity: 0.989	(60)
CNN-based diagnosis models for canine ulcerative keratitis	2019	CNN (GoogLeNet, ResNet, VGGNet)	281	Canine	Photograph	Accuracy: > 0.9	(55)
Detection of Cutaneous Tumours in Dogs Using Deep Learning Techniques	2019	CNN	1,500	Canine	Cytological image	Unperformed	(41)
Computer-Aided Diagnosis for Lung Lesion in Companion Animals from X-ray Images Using Deep Learning Techniques	2019	CNN	2,862	Canine Feline	Radiograph	Abnormal lung classification Accuracy: 0.723 Sensitivity: 0.81 Specificity: 0.637 Lung lesion detection Accuracy: 0.796 Sensitivity: 0.76 Specificity: 0.833	(32)
Comprehensive analysis of machine learning models for prediction of sub-clinical mastitis: Deep Learning and Gradient-Boosted Trees outperform other models	2019	CNN	364,249	Bovine	Dairy attributes/Sensor data	Accuracy: 0.84	(64)
Pilot study: Application of artificial intelligence for detecting left atrial enlargement on canine thoracic radiographs	2020	CNN	792	Canine	Radiograph	Accuracy: 0.827 Sensitivity: 0.684 Specificity: 0.871	(21)
Deep learning algorithms out-perform veterinary pathologists in detecting the mitotically most active tumour region	2020	RetinaNet (ResNet18) CNN (U-net, ResNet18, ResNet50)	32	Canine	Microscope slide image	Correlation coefficient: 0.963–0.979	(42)
Machine learning for syndromic surveillance using veterinary necropsy reports	2020	RNN (LSTM)	1,000	Unspecified	Necropsy report	F1 scores Gastrointestinal disease: 0.932 Respiratory disease: 0.947 Urinary disease: 0.752	(61)
Use of deep learning to detect cardiomegaly on thoracic radiographs in dogs	2020	CNN	1,465	Canine	Radiograph	AUROC: > 0.9	(20)
Deep feature learning for histopathological image classification of canine mammary tumours and human breast cancer	2020	CNN (VGGNet-16)	352	Canine	Histopathological image	Binary classification of canine mammary tumour accuracy: 0.93	(39)
Artificial intelligence evaluating primary thoracic lesions has an overall lower error rate compared to veterinarians or veterinarians in conjunction with the artificial intelligence	2020	CNN	22,120	Canine Feline	Radiograph	Overall error rate: 0.107	(17)
Using Deep Learning to Detect Spinal Cord Diseases on Thoracolumbar Magnetic Resonance Images of Dogs	2021	CNN	2,693	Canine	MRI	IVDP detection Sensitivity: 1 Specificity: 0.951 IVDE detection Sensitivity: 0.908 Specificity: 0.989 FCE detection Sensitivity: 0.622 Specificity: 0.979 ANNPE detection Sensitivity: 0.91 Specificity: 0.90	(33)
Histopathological Classification of Canine Cutaneous Round Cell Tumours Using Deep Learning: A Multi-Centre Study	2021	CNN (AlexNet, Inceptionv3, ResNet)	416	Canine	Histopathological image	Accuracy RCT classification: 0.917 Mast cell tumour grading: 1	(46)
Using Artificial Intelligence to Detect, Classify, and Objectively Score Severity of Rodent Cardiomyopathy	2021	CNN (ResNet50)	300	Rat	Microscope slide image	Spearman rank-order correlation between pathologist median grade and AI grade: 0.82	(43)
OncoPetNet: A DL based AI system for mitotic figure counting on H&E stained whole slide digital images in a large veterinary diagnostic lab setting	2021	CNN (ResNet18, UNet, EfficientNet, SE-Resnext)	3,845	Canine Feline	Haematoxylin and eosin-stained histologic slides	Improved mitotic count compared to human baselines	(47)
Computerised assisted evaluation system for canine cardiomegaly via key points detection with deep learning	2021	CNN (HRNet)	2,274	Canine	X-ray	Average performance: 0.864	(22)
Comparison of a Deep Learning Algorithm vs. Humans for Vertebral Heart Scale Measurements in Cats and Dogs Shows a High Degree of Agreement Among Readers	2021	CNN	60	Canine Feline	Radiograph	Intraclass correlation coefficient for vertebral heart scale between AI and specialists: 0.998 for both canine and feline	(18)
Disease Diagnosis of Dairy Cow by Deep Learning Based on Knowledge Graph and Transfer Learning	2021	CNN (KGTL_CNN)	21,649	Bovine	Medical records	F1 score for CNN based on knowledge graph: > 0.85	(58)
A deep learning model for CT-based kidney volume determination in dogs and normal reference definition	2022	nnUNet, UNETR	386	Canine	CT scan	R = 0.96 between manual voxel count and DL model	(30)
Developing a diagnosis model for dry eye disease in dogs using object detection	2022	CNN (YOLOv5)	95	Canine	Eye video image	mAP: 0.995	(57)
DL in veterinary medicine, an approach based on CNN to detect pulmonary abnormalities from lateral thoracic radiographs in cats	2022	CNN (ResNet50V2)	500	Feline	Radiograph	Accuracy: 0.82 F1 score: 0.85 Specificity: 0.75 Positive predictive value: 0.81 Sensitivity: 0.88	(31)
Cytologic scoring of equine exercise-induced pulmonary haemorrhage: Performance of human experts and a DL-based algorithm	2022	CNN (RetinaNet)	52	Equine	Microscope slide	Accuracy: 0.923	(44)
An automated deep learning method and novel cardiac index to detect canine cardiomegaly from simple radiography	2022	CNN (improved attention U-Net)	1,000	Canine	Radiograph	Left atrial & ventricular enlargement F1 score Vertebral heart score: 0.43 Adjusted heart volume index: 0.55	(19)
Deep learning-based diagnosis of feline hypertrophic cardiomyopathy	2023	CNN (Resnet50V2, Resnet152, InceptionResnetV2, MobilenetV2, Xception)	273	Feline	Radiograph	Accuracy: > 0.9	(23)
Deep learning-based diagnosis of stifle joint diseases in dogs	2023	CNN (R-CNN, ResNet)	2,382	Canine	Radiograph	Accuracy: > 0.8	(53)
Canine Mammary Tumour Histopathological Image Classification via Computer-Aided Pathology: An Available Dataset for Imaging Analysis	2023	CNN (VGG16, InceptionV3, EfficientNet)	1,056	Canine	Haematoxylin and eosin-stained histologic images	Accuracy: 0.63–0.85	(49)
Automated diagnosis of 7 canine skin tumours using machine learning on H&E-stained whole slide images	2023	CNN (EfficientNet B5)	350	Canine	Haematoxylin and eosin-stained histologic images	Accuracy: ~0.95	(50)
Application of convolutional neural network for analysing hepatic fibrosis in mice	2023	CNN (Xception)	33	Mice	Whole slide images	Correlation with pathologist hepatic fibrosis grade (*r* = 0.9067)	(52)
Histological classification of canine and feline lymphoma using a modular approach based on deep learning and advanced image processing	2023	CNN (Unet++)	116 Canine 38 Feline	Canine Feline	Haematoxylin and eosin-stained histologic images	Accuracy: 0.92 for canine 0.84 for feline	(45)
Automatic grading of intervertebral disc degeneration in lumbar dog spines	2023	CNN (VGG16)	5,991	Canine	MRI	Accuracy: > 0.9 Sensitivity: > 0.83 (except 1 class)	(26)
Use of deep learning for the classification of hyperplastic lymph node and common subtypes of canine lymphomas: a preliminary study	2024	CNN (GoogLeNet)	1,530	Canine	Whole slide images	Accuracy: 0.99	(51)
Automatic classification and grading of canine tracheal collapse on thoracic radiographs by using deep learning	2024	CNN (YOLOv3, YOLOv4, YOLOv4 tiny)	600	Canine	Radiograph	Accuracy: 0.989 Sensitivity: 0.983 Specificity: 0.992	(27)
Deep learning-based ultrasonographic classification of canine chronic kidney disease	2024	CNN (YOLOv8-n)	883	Canine	Ultrasound	Accuracy: 0.46	(25)

## Results

The articles that met the inclusion criteria were initially arranged into applications of DL in diagnostics and other domains. Next, in the diagnostics section, articles that were relevant to the diagnosis of diseases were summarised and grouped based on the type of images and/or data they investigated. This helped to synthesise the relevance of these techniques in accordance with the wide variety of information required for accurate diagnosis in the veterinary health context. The remaining section included research on DL applications in areas outside of diagnostics but still consistent with the theme of detecting or predicting diseases.

**Figure 3:**
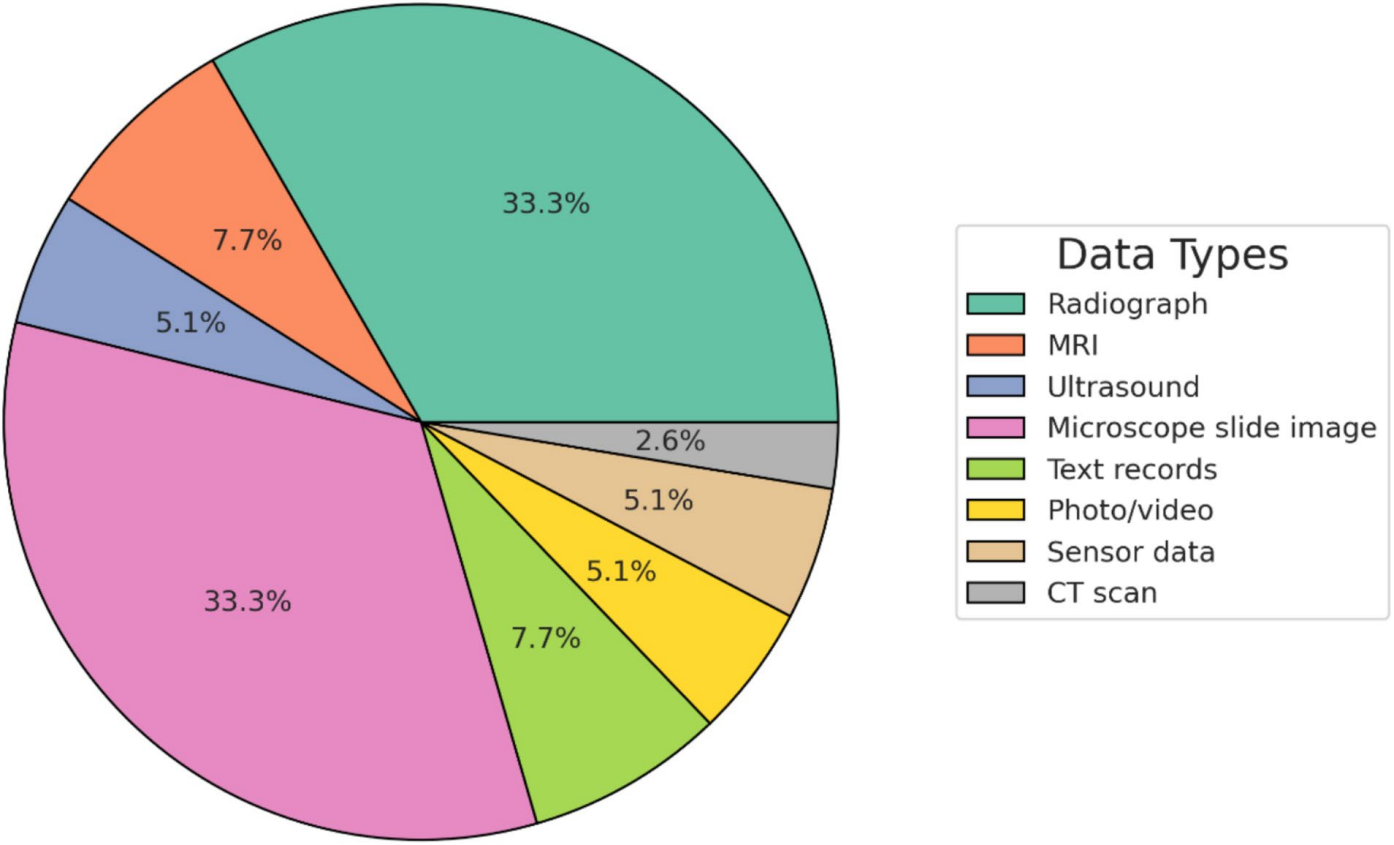
The proportion of different data modalities used in the deep learning studies.

**Figure 4:**
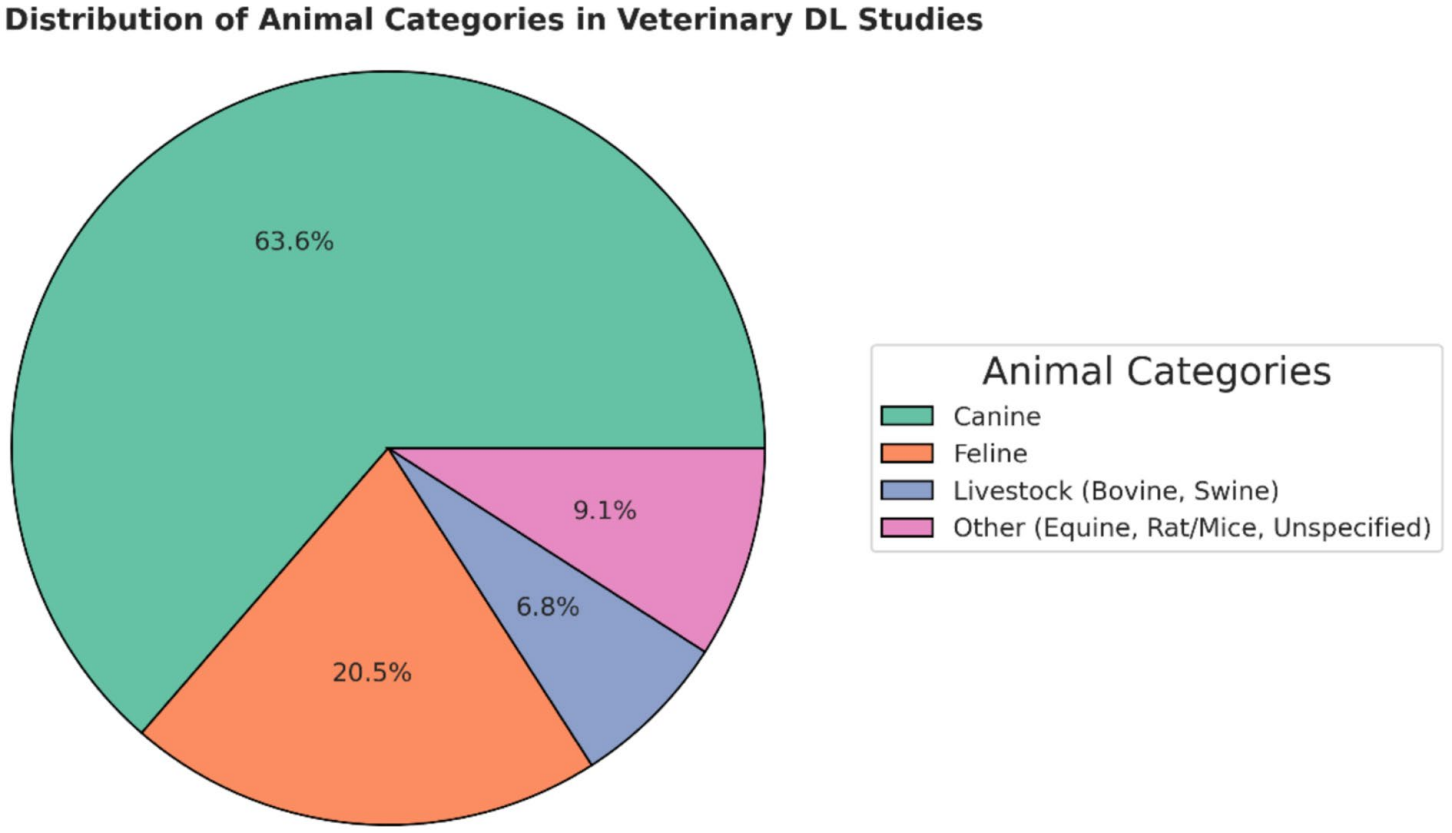
The proportion of different species researched in the deep learning studies.

**Figure 5:**
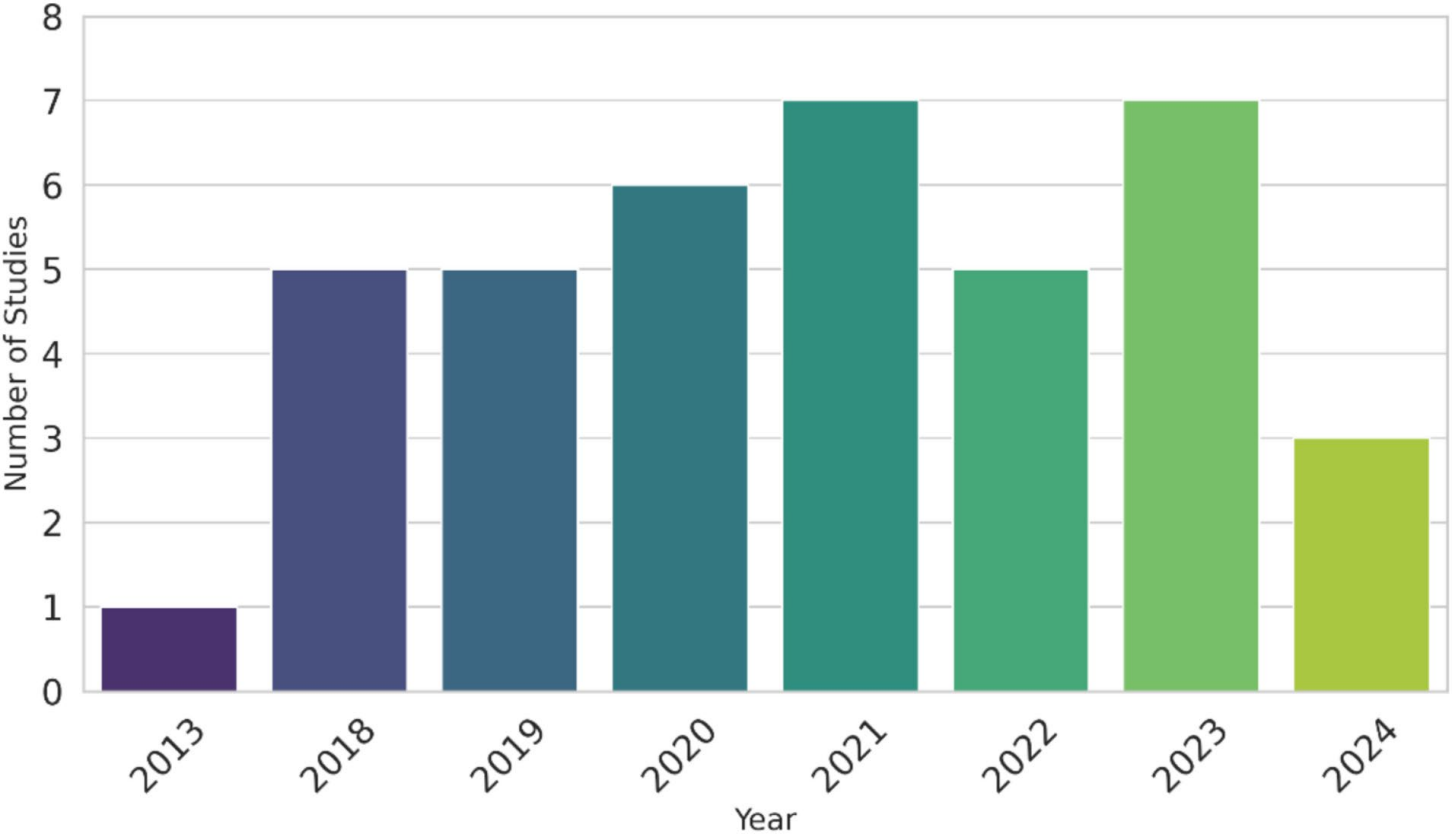
The number of deep learning studies in veterinary medicine across the years.

Most of the diagnostic DL research in veterinary medicine is related to the interpretation of medical images. The proportion of different data types used in the DL studies is presented in Figure 3, and the species-wise studies in Figure 4. Most of the DL studies (84%) were on canine (64%) and feline (20%), highlighting the gap in research on other animals, especially those in the livestock industry. The increasing development of DL within the veterinary health/diagnostics context since its inception in 2013 is presented in Figure 5.

Over the years, the quest for improved performance in various studies led to the exploration of different methodologies. When examining the trajectory of accuracy over the years, no clear overarching trend emerged. It appeared model accuracy was dependent on the problems these studies attempted to solve, the animal species, the size of the dataset available, and the complexity of the models utilised. While there was not a consistent upward trend in the size of training data across all studies, variations were notable depending on the domains. Datasets for diagnostics studies involving canine and feline companion animals were larger compared to those involving other animals, which indicates limited research on other animals, such as horses, as DL modelling required larger data sizes for better classification performance. Moreover, there was a discernible disparity in sample sizes, with text/health records and sensor data studies often featuring more substantial datasets compared to studies using radiograph data. Finally, there was not a dramatic shift in the types of models used over the years. CNNs continued to be the predominant model type in various studies, as seen in Table 1, which suggested that the research in DL was still at an early stage, as other DL models were rapidly evolving and yet to be investigated in the veterinary health context. However, a noteworthy observation was the incorporation of transfer learning techniques based on more updated sophisticated models, such as different ResNet versions throughout the years reviewed. The use of transfer learning, a technique which enhances model performance by leveraging pre-existing knowledge from a pre-trained model often on larger datasets of images, and fine tuning it for a new image classification task allows the model to adapt effectively to the specific contexts found in the domain of animal health (15). The following sections examine how deep learning have been applied across key domains of veterinary practice.

### Deep learning involved in disease diagnosis

#### Veterinary medical imaging

Traditional ML strategies can be used for medical image analysis, such as the bag of features (BoF) strategy that was applied by Yoon et al. (16), which aimed to distinguish between normal and abnormal radiographic findings from canine thoracic radiographs across many different regions. The same study included a DL component using a CNN to accomplish the same goal. A direct comparison between the two strategies in this study showed that CNN had higher accuracy and sensitivity measures in performing these tasks when compared to BoF (16). Similarly, a comprehensive study trained a CNN model using a large sample of 22,000 veterinary radiographs of cats and dogs combined to predict and identify 15 types of primary thoracic lesions from the radiographs. It showed that classification based on DL produced a significantly lower error rate when compared to the classification performance made by veterinarians (17). Interestingly, the study also asked veterinarians to make predictions with access to the results provided by the DL model, but the experts’ prediction results did not improve significantly, which was implied by the authors that experts have a certain level of scepticism about the results of the AI technology. Thoracic radiographs were also analysed to quantify cardiac enlargement to predict cardiac diseases, where DL modelling achieved high concordance between its assessment and human specialists across both canine and feline patients (18). This success was mirrored in another study, where a DL model’s cardiac index calculation outperformed the clinical standard produced by veterinary radiologists in predicting cardiac enlargement (19). Expanding on these applications in thoracic radiographic analysis, convolutional neural networks (CNNs) have been particularly effective in detecting cardiomegaly (heart enlargement) from these images, achieving high diagnostic accuracy in canines (20, 21). Further advancements in DL have led to the creation of cardiac scoring models for predicting and diagnosing canine heart diseases (22). CNN models have also been applied to feline heart conditions, such as feline hypertrophic cardiomyopathy, achieving diagnostic accuracies exceeding 90% in identifying the disease from radiographic images (23). These findings highlight the adaptability of DL techniques across different species for the diagnosis of heart diseases.

Further illustrating CNN’s utility in image analysis, Banzato et al. (24) showed that CNNs outperformed non-invasive diagnostic tests, such as serum biochemistry and cytology, in the detection of hepatic diseases from ultrasound images, underscoring DL’s potential to enhance diagnostic confidence. However, a separate study on canine chronic kidney disease (CKD) highlighted ongoing challenges, particularly in tackling more complex multi-class classification problems. The CNNs studied struggled to classify five stages of the disease from ultrasound images, achieving a performance accuracy of only 0.46 on average, likely due to the subtle differences between stages and the limitations of the ultrasound imaging. Importantly, ultrasound may not be the most suitable modality for accurate CKD staging, which affects the reliability of the ground truth used to train and evaluate the model. Since DL models are highly dependent on the quality of their training data, a suboptimal ground truth can constrain model performance, regardless of architectural improvements. This underscores the importance of carefully selecting diagnostic modalities and ground truth definitions in AI studies to ensure meaningful and clinically relevant outcomes. While model selection and hyperparameter tuning remain critical, optimising study design and ensuring high-quality, confirmatory diagnostic data are equally essential for unlocking the full potential of DL applications in veterinary medicine (25).

VGG (visual geometry group) networks have been applied successfully to MRI scans of canine lumbar discs, where they were used to grade intervertebral disc degeneration, achieving over 0.9 accuracy across all five degeneration grades (26). Another noteworthy development in DL for veterinary imaging was the use of YOLO (You Only Look Once) a CNN based model. Originally designed for object detection, YOLO v3, v4, and v4 tiny have been adapted to not only detect object but also classify tracheal collapse grades (Normal, grade 1–2, and grade 3–4) from lateral cervicothoracic canine radiographs, offering a versatile tool for both detection and classification tasks (27).

CNN techniques have developed rapidly and branched off into more specialised networks with addition of image segmentation components to better analyse medical images (28). Image segmentation divides the image into multiple significant parts to make the input dataset more informative to analyse (29). This technique was investigated in a kidney disease diagnosis study where a Unet CNN with an image segmentation component was tested to successfully estimate kidney volume from CT scans (30). Similarly, inbuilt image segmentation in a CNN was used to detect pulmonary abnormalities in feline radiographs, showcasing its utility (31). This level of diagnostic success was not consistently reproduced in other studies such as in the diagnosis of lung lesions in both dogs and cats from x-ray images, the diagnostic accuracy was only around 70–80% (32). Another study demonstrated low levels of performance for the detection of neoplasms (sensitivity ranged from 0 to37.5%) and syringomyelia (sensitivity ranged from 0 to 10%) on MRI images using custom CNN models. The poor performance was due to the limited training samples of those cases (33), demonstrating that in certain contexts, DL remains an evolving technology requiring further refinement. Additional experimentation in both modelling approaches and pipeline design is necessary to optimise the algorithmic efficacy.

In another MRI analysis study, the authors attempted to distinguish between meningioma and glioma conditions in dogs from a selection of MRI images utilising a process known as transfer learning to develop a CNN model from a pre-trained GoogLeNet CNN (deep neural network consisting of 144 layers) (34). The GoogLeNet has been trained on the ImageNet database with up to 1.2 million images across 1,000 categories to extract the CNN features. It was retrained on a new dataset of MRI images which achieved high accuracy (91 and 94%, respectively) of the model in correctly classifying the condition from both pre-and post-contrast MRI images. The correct differentiation between the two conditions is necessary for choosing the right treatment procedures that could lead to better health outcomes for patients. Retraining pre-trained models reduces the computational resources and potentially the number of images required to apply DL, increasing the accessibility of AI’s advantages in various animal health applications. It is still important to note that this improvement in accessibility may come in exchange for accuracy in certain situations.

Artificial neural network (ANN) is another DL technique that is composed of fully connected layers where each neuron is connected to every neuron in directly neighbouring layers, which are more commonly used for general purpose problem solving (35). It was applied to identify canine hip joints on ventrodorsal pelvis radiographs with low classification error, and high sensitivity and specificity measures of 8.9, 86, and 100%, respectively (36). ANN has the flexibility to choose different activation functions for nonlinear function learning purposes and change the number of hidden layers and nodes in these hidden layers, to improve its performance to suit various image processing needs (37). An updated study instead used a deep CNN for the detection of the hip joint and extended their aim to the classification of hip dysplasia from pelvis radiographs in two stages. The first stage involved identifying the boundary box of the hip joint from the radiographs using the YOLOv3 object detection algorithm (38). These regions were then cropped and put through the second stage of analysis, where a CNN model graded hip dysplasia, which resulted in a high specificity of 0.92 for FCI scores in the “C-E” group. However, the model’s sensitivity metric was low at 0.53, suggesting its failure to identify many positive cases (false negative). One contributing factor to this limitation was that the image dataset contained unbalanced annotated images, where certain types of hip dysplasia were under-represented, which impaired the performance of the model in its testing phase (38). This outlines the importance of having a large data set to train an effective CNN.

#### Microscope slide images

Microscopic examination of tissues, cells, and blood on microscope slide images is known to be tedious and challenging for disease diagnosis, even for well-trained specialists (39). DL techniques, particularly CNN, have proven effective in addressing these challenges, such as its utilisation in recognising reticulocytes in cat blood smears to a high accuracy of 98.7% (40). Another study used CNN modelling to diagnose and classify abnormal cell growth in canine skin samples from cytological images (a subset of microscope slide images), improving cancer detection (41). Furthermore, DL methods, particularly CNNs, outperformed veterinary pathologists in grading prognostic elements of canine tumours based on stained canine cutaneous mast cell tumours (42). Along with the results, it was noted that the chosen section of the slide images for analysis by veterinary pathologists was quite varied, thus producing more inconsistent results between experts in their mitotic counts. Also, a more advanced network based on the CNN known as ResNet 50 was able to match with pathologists’ grading of cardiomyopathy severity from microscope slide images in rodents with a Spearman rank-order correlation of 0.82 (43). In some cases, DL techniques surpass human performance as indicated by a study on horses, where the CNN outperformed human specialists (76% accuracy) in diagnosing exercise-induced pulmonary haemorrhage, achieving a high accuracy of 92% (44). These studies emphasised the utility of DL in diagnosing diseases across different animals, showcasing the potential of these techniques to match or even outperform human specialists in certain contexts within veterinary medicine.

Recent advancements have adopted a more flexible approach to the analysis of microscope slide images, such as using DL models to first preprocess the images by dividing them into grid sections to ensure areas of interest are separated from irrelevant backgrounds. This step is crucial for achieving high classification accuracy, particularly when applying segmentation techniques like Unet, which enables the identification of relevant regions prior to classification (45), and combining different parts of the algorithm from various known architectures to suit different needs like the aggregate model used in this study, where different CNNs (AlexNet, Inception v3, and ResNet) are combined to form the ARCTA algorithm, which was utilised to accurately classify canine cutaneous round cell tumours (accuracy of 91.7%) and mast cell tumours (accuracy of 100%) (46). This approach of utilising aggregate modelling have also shown success in mitotic figure count, enabling critical early tumour detection (47).

Pretrained models like VGGNet-16, have been applied to classify canine mammary tumours and human breast cancer from histopathological images, achieving improved accuracy when combined with traditional ML classifiers (93%) (39). The incorporation of transfer learning helps to alleviate the issues of using a small image dataset by only fine-tuning the model parameters based on the knowledge obtained from a large dataset (48). Recent studies have leveraged advanced CNN architectures such as VGG16, InceptionV3, and EfficientNet as feature extractors for canine tumour histopathology. By removing the final classification layers of these networks and feeding their outputs into traditional machine learning algorithms like support vector machines (SVM), researchers have enhanced differentiation between benign and malignant tumours (49). In some cases, transfer learning-based approaches have demonstrated particularly high accuracies, such as the use of EfficientNet B5, which achieved approximately 95% accuracy in classifying seven different types of canine skin tumours, though this still fell short of human expert performance levels (50). Another study used GoogLeNet transfer learning to classify three classes of canine lymphoma from whole slide images, achieving 99% accuracy in the test set (51). Similarly, XceptionNet was employed to analyse whole slide images in mice, where it showed a strong correlation (*r* = 0.9067) with pathologists’ grading of hepatic fibrosis (52), further illustrating the viability of DL techniques in diagnostic contexts.

Another example where combining different machine learning techniques presents a promising avenue in the veterinary health context. The R-CNN method, an object detection method, was first utilised to extract regions of interest then a ResNet classification model was utilised to detect patterns related to canine stifle joint disease with an accuracy over 80% (53). These results demonstrate the potential of combining multiple CNN networks for improved classification performance in veterinary medicine.

#### RGB images

The widespread availability of RGB images from smartphones and digital cameras has made DL models more accessible, highlighting potential beneficial applications and ease of access to this technology in veterinary medicine (54). Hundreds of annotated photo images of canine eyes were trained and evaluated with CNN models: GoogleNet, ResNet, and VGGNet, to determine and predict corneal ulcer severity in dogs (55). It was shown that many of these DL models achieved accuracies beyond 90% for identifying the different levels of corneal ulcer severity. Another study supports the above claim as its dataset was formed from photo images of equine eyes taken via smartphones (56). Four different CNN models (MobileNetV2, InceptionV3, VGG16, VGG19) were studied to classify 3 categories of eye conditions with a particular focus on equine uveitis (a particular eye inflammatory disease), the top performing model achieved a validation accuracy of 96% (56). A part of the imaging data of the eye was collected using a smartphone camera, which further highlights the applicability of DL algorithms in the analysis of photo images gathered via smartphones in the animal health domain. DL techniques have performed well deciphering limited-quality smartphone image data to produce highly accurate results. Images can also be obtained from videos by using object detection DL algorithms to form the image dataset for analysis. In a separate study on canine eye disease, images were isolated from video footages of the face of the animals for application in DL models (57).

#### Text analysis

DL can be used to extract textual information to produce diagnostic suggestions. Disease knowledge was first extracted from a dairy cattle disease graph found in literature, as well as information obtained from experts and features found in medical records on a variety of dairy cow diseases such as mastitis, forestomach atony, rumen indigestion, gastroenteritis, rumen acidosis and abomasum dislocation to form the initial dataset. Then a CNN model was pretrained on this initial dataset to obtain the model parameters and weights. Finally, a transfer learning technique was employed utilising this pretrained model on a more limited separate real-life dataset of textual features of the above outlined dairy cow diseases for training and testing to ascertain the diagnostic performance of the developed DL approach (58). The developed model showed a promising F1 score around 86% in the automation of the diagnosis of dairy cow diseases. The authors compared this model’s performance to other standalone ML models, including support vector machine, random forests, and decision tree, as well as DL methods: recurrent neural network (RNN) and CNN. The results showed the effectiveness of the transfer learning strategy with a pretrained model.

RNN is a DL method which is devised to formulate sequential patterns such as texts and videos (59). RNN was adopted for detection of chronic cat kidney diseases from historical electronic hospital records of patients and achieved high classification performance of 0.907 and 0.989 for sensitivity and specificity, respectively (60). In other text-based veterinary medicine data like necropsy reports as seen in the study performed by Bollig et al. (61), an RNN with a long short-term memory (LSTM) design successfully classified evidence of gastrointestinal, respiratory and/or urinary diseases, which helps to reveal the features of these diseases such as its epidemiological nature.

#### Sensors

DL has been used to predict disease occurrence in animals based on historical sensor data. One study utilised a recurrent neural network (RNN) to generate an autoencoder that recognised environmental sensor data, such as CO2, temperature, and humidity, which were associated with housing environment health for pigs (62). The collected sensor data was evaluated by the GRU-autoencoder based on its similarity to normal data. If the data exceeds a specific anomaly threshold, optimised using Particle Swarm Optimisation, the algorithm outputs a prediction warning for respiratory disease. As sensor data is collected in real-time, it allows timely prediction for farmers to act and intervene to prevent the development of respiratory disease. Another type of data which is collected routinely is in the field of dairy, where milk attributes are continuously being collected and recorded by machines at each milking. An important reason for this is to monitor for the occurrence of mastitis in the dairy cows, as contamination will affect the milk quality and cause it to be discarded. Traditionally, statistical methods have been used to predict subclinical mastitis from dairy-related attributes (63). However, one recent study utilised a relatively simple multilayer feed-forward deep neural network to forecast subclinical mastitis with a high accuracy of 84% based on multiple milking variables, showcasing the capabilities of DL techniques in this field (64).

From the literature, it appears that there is more research being performed on inferring, predicting, and tracking animal behaviour from sensor data, where it found moderate to higher levels of success (65, 66). Compared to this, the analysis of sequential sensor data using DL algorithms appear to be premature at this current stage of development in the diagnosis of diseases in animals. Thus, more research in this area may uncover new and more comprehensive information that can be used to inform disease management and prevention in livestock farming.

### Issues for consideration when designing and conducting studies

Accountability and ethical considerations in AI assisted veterinary practice: The technology to replicate human thought processes in AI analysis is still evolving. The tasks assigned to AI algorithms are diverse and complex, requiring tailored infrastructure for each study (10). For example, Vinicki’s study required a precise image scoring system consistent with expert evaluations to correctly label images and guide AI in accurately classifying reticulocytes (40). This underscores the importance of expert consultation throughout the design and implementation phases, ensuring results are interpreted with specialist input for meaningful understanding.

Many studies reviewed here indicate that DL models are most effective when used as decision-support tools rather than standalone diagnostic systems. While accurate and reliable AI applications can enhance patient outcomes, unreliable AI use may introduce risks, particularly in veterinary medicine, where training datasets are often limited. Unlike human medical AI, which benefits from vast, standardised, and well-validated datasets, veterinary AI faces challenges due to species-specific variability and context dependent variables (67). Companion animals such as dogs and cats are more commonly studied, while data for exotic and livestock species remain scarce as shown in this review. In addition, animal health data are often lower in quality, unstructured, inconsistently collected across institutions, and subject to less standardisation and scrutiny compared to human medical datasets (68). These factors significantly impact training quality, as DL models rely heavily on well-curated, high-quality datasets to achieve precise and accurate predictions. Consequently, data imbalance and variability affect model generalisability, resulting in an AI system trained on feline or canine images that may not be directly transferable to other species, even when diagnosing the same disease. This underscores the critical role of veterinarians in validating AI-generated outputs and ensuring that AI is used responsibly as a supporting tool, rather than as an independent diagnostic system (69). Proper clinical oversight is essential for ethical and effective AI integration in veterinary medicine.

### Sample size and data quality

A large volume of training data is crucial for developing and testing reliable DL applications (70). Although specific sample size requirements vary by context, certain factors can help estimate the sample size required for effective modelling (71). The size and complexity of the model significantly impact the required dataset, as demonstrated in Krizhevsky’s study where training a deep CNN model required over 1 million labelled images (72). Although not directly mentioned, it can be inferred that the number of predicted classes affects the required sample size, given the study’s aim to classify 1,000 different classes. In contrast, veterinary medicine often focuses on predicting fewer classes, with currently annotated and labelled data being limited, especially within specific veterinary imaging cases, where small sample sizes and unbalanced classes are common limitations (73). Veterinary AI studies often have smaller sample sizes due to factors such as having smaller and more dispersed patient (companion and livestock) populations, which makes data collection difficult as veterinary records are often heterogeneous (74). In addition, species-specific variability requires separate datasets for different animals, further fragmenting available data for prospective studies. Hence sourcing animal samples that meet specific disease criteria is big challenge, often necessitating retrospective data collection from historical health records, which may contain missing or inaccurate information (75). However, increasing sample size alone is not always beneficial if the data quality is compromised. If poorly labelled, inconsistent, or inaccurate ground truth data are used, AI models may exhibit misleading performance gains while lacking true clinical utility. As veterinary AI research progresses, larger and more diverse datasets will be needed, but they must be carefully curated, standardised, and validated to maintain model reliability (76). In human medicine, one approach to addressing small sample sizes is the use of pre-trained large models, which can be fine-tuned for specific tasks while leveraging existing large scale datasets (77). This is known as transfer learning, which is a promising approach that offers significant potential for further exploration and application in the field of animal health, particularly in settings where data availability is constrained. Data augmentation is another potential solution as it is a method used to enhance the variety of the training dataset by applying various transformations to the existing data, producing altered versions that remain representative of the original dataset (78). This artificially inflates the dataset and increases the effective sample size for the training of the model, which can help to teach AI models more diverse features aiding in its classification performance.

### Evaluation and validation challenges

Ensuring the reliability and generalisability of DL models is highly dependent on the validation strategies employed. While a clean and well-structured dataset is fundamental, models must undergo a rigorous validation process to confirm that they can perform beyond the controlled environment in which they were trained. Without appropriate validation, even highly optimised models may exhibit overfitting, suffer from dataset biases, or fail to generalise to real-world clinical applications, limiting their practical use (79). One of the major challenges in veterinary AI research is the lack of universally standardised and fully labelled validation datasets, which complicates fair comparisons between different AI models. Unlike human medical AI, where large-scale benchmark datasets exist for model evaluation, veterinary AI studies often rely on institution specific datasets, making it difficult to directly compare performance across different studies. This absence of standardisation can lead to inflated performance metrics if validation datasets do not accurately represent real world conditions.

The selection of validation strategies is therefore crucial. Internal validation, where models are assessed using the same dataset on which they were trained, is an essential initial step but does not account for variations in clinical practice, different imaging modalities, or novel cases encountered in real-world settings (80). External and clinical validation are necessary to test the model’s performance across diverse datasets, imaging techniques, and patient populations. Without these steps, a model that appears to perform well under controlled conditions may fail when deployed in practice. Additionally, the quality of validation datasets plays a significant role in model reliability. If datasets are small, unbalanced, or inconsistently annotated, AI models may demonstrate misleading performance gains while lacking true clinical utility. Poor validation design can also lead to circular reasoning, where models inadvertently learn dataset-specific artefacts rather than true disease characteristics.

Given these challenges, AI studies should transparently report their validation methodologies to allow accurate interpretation and comparability across research. Standardising validation frameworks for veterinary AI (81), including establishing common datasets and benchmarking protocols, would be a critical step in improving model assessment and ensuring AI applications are both scientifically rigorous and clinically relevant. Despite the necessity of these validation steps, there remains a lack of regulatory validation strategies that apply to all DL modelling, particularly in veterinary applications. Again, unlike in human medical AI, where regulatory frameworks are more established, veterinary DL models are often developed with less stringent oversight. This gap in regulatory validation could lead to risks for animal patients if veterinary experts are not actively involved throughout the entire model development lifecycle, including post-implementation monitoring and error reporting (82).

### Data analysis workflow

An effective and smooth analysis pipeline is key to the successful and efficient building of DL networks. General principles to follow include data preprocessing techniques like data augmentation, normalisation, and class imbalance handling to ensure the model robustness (83). It is crucial to understand the characteristics of the images and provide accurately annotated data for processing. Lapses in these areas can lead to poor performance or unexpected results. Choosing the most appropriate model is essential, considering factors like the number of classes and problem complexity. Network architecture design and careful selection of hyperparameters, such as the loss function, optimisation algorithm, and learning rate, significantly affect model convergence and performance (83).

### Black box approach

DL methods are often called “black-box” approaches due to their complex and opaque inner workings, making them difficult to interpret. None of the reviewed veterinary health studies attempted to address this issue in a technical fashion, highlighting the nascent stage of DL research in this field. However, explainable AI (X-AI) approaches, such as GradCAM, which generates gradient maps, and LIME (Local Interpretable Model-agnostic Explanations), which explains how input modifications affect model predictions, are used in human medical image analysis (73). Researching and implementing X-AI in veterinary medicine could enhance understanding and improve imaging diagnostics. Beyond technical interpretability, transparency is also a critical consideration. Veterinary users need to understand and trust AI driven tools before integrating them into clinical practice (67). A lack of transparency in how these models generate predictions may hinder their adoption, reinforcing the need for X-AI methods to enhance trust and usability in veterinary diagnostics. Researching and implementing X-AI in veterinary medicine could improve both interpretability and confidence in AI-assisted imaging diagnostics.

### Opportunities for future DL applications in the animal health domain drawing inspiration from human health and other domains

DL techniques in human health can guide and indicate the potential for veterinary medicine. Application of AI image analysis for detecting skin tumours, bone fractures, and lung infections in humans could be similarly developed and adapted for diagnosing animal diseases (84). The application of transfer learning in veterinary medicine for these types of disease classification is a potential avenue for exploration as these types of disease research are currently limiting in the animal health context.

AI can transform healthcare delivery by improving efficacy, accessibility, and personalisation. For example, human healthcare providers utilise monitoring devices and smart phone technology to obtain real-time patient vitals for monitoring purposes (85). This area could be adapted to track animal health indicators, enhancing veterinary health management. Virtual assistants with NLP capabilities, which provide health and medication information post-hospital visits, have shown improved patient outcomes and could similarly benefit animal healthcare, particularly in rural and underserved areas. NLP techniques that extract information from human clinical records can also be applied to veterinary health records for clinical research, revealing additional information to support veterinarians (86). Even though this type of research is still relatively nascent in human medicine but as this technology improves, it could potentially be implemented in the analysis of veterinary health records to support better decision making by veterinarians. In another aspect of medicine, CNNs have been used to track and analyse surgical procedures to assess surgeon performance during medical training (87). This application holds promise in veterinary medicine for training new surgeons and providing individualised feedback to assist and support veterinarians in training.

Human medicine has successfully utilised specific models such as BERT for natural language processing and ResNet for image classification, achieving high accuracy and robustness (88, 89). These successes are partly due to the availability of large, diverse datasets and synthetic data generation techniques, which help augment training datasets and improve model performance. In contrast, veterinary medicine lacks similarly extensive datasets and established models, making it a prime candidate for applying and adapting these advanced techniques. The success of synthetic data in human studies, enhancing model training and performance, suggests a promising avenue for veterinary applications, potentially mitigating the challenges posed by limited real-world data.

### Rapid development in AI

AI is advancing at an unprecedented rate, with new models continually emerging in human medical applications. A recent study published by Google introduced Med-PaLM, formerly known as MultiMedQA, a comprehensive benchmark for evaluating the clinical knowledge of large language models (LLMs) across various medical topics (90). While Flan-PaLM, a 540-billion parameter LLM, achieves state-of-the-art accuracy on Med-PaLM datasets, human evaluations reveal key shortcomings in areas such as comprehension and reasoning, underscoring the need for improved evaluation frameworks and methodologies to make LLMs safe and useful in clinical settings (91). Med-PaLM and its evaluation framework hold significant potential for adaptation in veterinary medicine. By tailoring these benchmarks to veterinary-specific datasets, it is possible to assess and enhance the accuracy and safety of LLMs in diagnosing and treating animal health conditions ensuring that LLMs can effectively support veterinary professionals.

The recent introduction of Med-Gemini, a family of highly capable multimodal models specialised in medicine, highlights the rapid development in AI. Med-Gemini models excel in advanced reasoning, up-to-date medical knowledge access, and complex multimodal data understanding. It achieved state of the art performance on 10 out of 14 medical benchmarks (92). Med-Gemini’s capabilities to interpret and analyse complex data could be adapted to diagnose animal health conditions using diverse data sources, including images, health records, and sensor data. Moreover, its capability to surpass human experts in medical text summarisation and video question answering could enhance veterinary training and decision-making processes. These strengths suggest promising potential for the applications of these tools in veterinary medicine, providing more accurate and comprehensive diagnostic tools and improving overall animal healthcare.

The adaptation of transformer models, initially developed for natural language processing, has opened new frontiers in veterinary computer vision applications. The “transformer” architecture, based on a self-attention mechanism, allows the model to weigh the relative importance of different features independently of their order, providing a more flexible and nuanced approach to data interpretation (93). This approach has demonstrated comparable performance to CNNs in image classification tasks (94). In a recent medical imaging study, a Vision Transformer (ViT) was used to analyse PET brain scans, classifying healthy tissue versus Alzheimer’s disease, and outperformed the CNN-based VGG19 (95). In oncology, ViT models have shown superior performance in classifying skin cancer from lesion images (96). Another study highlighted ViT’s potential in detecting tuberculosis in chest X-rays, where a hybrid approach incorporating a ViT component with a CNN backbone achieved higher classification performance than a standalone CNN (97). These successes suggest that researching ViTs in veterinary health contexts could improve diagnostic accuracy in medical imaging, which is crucial for effective treatment and disease control in animals. Moreover, generative adversarial networks (GANs) and variational autoencoders (VAEs) showed promise in mitigating the need for extensive labelled datasets in medical image analysis (98). By synthesising realistic medical images, these models can augment training data, potentially enhancing the performance and generalizability of DL models even with limited real-world samples (99). Such approaches could be useful in veterinary diagnostic where limited images are currently available. In summary, the rapid development of AI technologies, exemplified by models like Med-Gemini, Med-PaLM, ViTs, GANs, and VAEs, presents significant opportunities for advancing veterinary medicine. By adapting these technologies and benchmarks to veterinary contexts, the field can benefit from improved diagnostic tools, more robust data augmentation techniques, and ultimately, better health outcomes for animals.

## Conclusion

This review has highlighted the application of DL in veterinary medicine. This is a rapidly evolving area of research with increasing attention on its use in the veterinary healthcare industry in recent years. Its advantages are better understood and can be utilised to benefit many aspects of the industry as seen in the examples discussed, particularly in image analysis, which is enabling health specialists and farmers to develop optimal and timely treatment and prevention plans for the best possible health outcomes for affected animals.

The creation of training datasets for veterinary diagnostics is both labour-intensive and costly, presenting a significant bottleneck in the broader application of AI within this field. The limited availability of comprehensive datasets, encompassing various diagnostic modalities, further constrains the successful deployment and optimization of AI-driven tools in veterinary diagnostics.

This review underscores the urgent need to create standardised, high-quality large training datasets that include a wide array of diagnostic modalities and animal species. Given the inherent diversity of species within veterinary practice, fostering international collaboration is not only advantageous but also essential for the successful implementation and fine-tuning of AI models in veterinary diagnostics. To facilitate this crucial endeavour, we suggest the formation of an international consortium focused on veterinary phenomics for AI. Such a collaborative framework would not only accelerate the assembly of comprehensive and interoperable datasets but also catalyse advancements in AI-driven veterinary diagnostic techniques, thereby elevating the quality and efficacy of animal healthcare globally.

## Data Availability

The original contributions presented in the study are included in the article/supplementary material, further inquiries can be directed to the corresponding author.
